# Achieving diversity, inclusion and equity in the nursing workforce

**DOI:** 10.1590/1518-8345.0000-3254

**Published:** 2020-02-14

**Authors:** Brigit Carter

**Affiliations:** 1 School of Nursing, Duke University, North Carolina, United States of America.


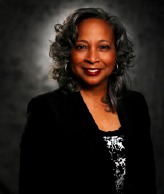


Research has shown that individuals from minority groups in the United States have inferior health, experience added problems accessing care, have an increased likelihood of being uninsured and even when insured will more often receive lower quality health care than non-minorities. Maternal and child health, cancer screening, diabetes and access to care are examples in which disparities persist in the minority and low-income populations^(^ 1 ^)^. There are some indicators that there are signs of improvement in the quality care for some groups, however the quality measures of disparities related to age, race/ethnicity, and income have not demonstrated the same levels of improvement^(^ 2 ^)^. 

Nursing has the capability to be the leading force in reducing health inequities. Increasing the racial and ethnic diversity of the nursing workforce has been proposed as a significant strategy to bring equality into health care access and delivery and there is strong supportive evidence across the United States to support this assertion. While cultural proficiency is important for all nurses to attain, the importance of an ethnically and racially diverse nursing workforce remains. Racial and ethnic minorities are more likely to return to and serve their underrepresented communities, bridge the cultural and linguistic gaps inpatient education, and provide a broad and different cultural prospective to all conversations within nursing^(^ 3 ^)^. The positive impact of increasing nursing workforce diversity reverberates across education, research and clinical areas. 

## Education

The role of academia in addressing the nursing workforce diversity is multidimensional. As an institution seeks to improve diversity and inclusion efforts, it seems appropriate to define diversity and inclusion within that community and/or environment. While there are standard definitions for diversity, it should be articulated what the expected outcomes are by achieving diversity and how diversity will be supported once this new and diverse community has been developed. Building an inclusive environment must often come before the goal of diversification. In order to create an inclusive environment, is critical to establish processes that nurture difficult conversations, address mindsets towards diversity and develop training/educational resources which support and enhance our ability to create an inclusionary culture. 

The conditions academia creates should strengthen all student’s knowledge of how to assess vulnerable populations for social determinants, disparities and inequities. The curriculum should include foundational tools that will promote the student’s development and ability to address health disparities through leadership development and health policy mechanisms. Pipeline programs which seek to identify and support underrepresented minority faculty and students should be created and integrated into the fabric of the school. With the development of pipeline programs, it is imperative to address barriers that commonly hinder the admission, matriculation and successful graduation of underrepresented students by developing academic, financial and mentorship support interventions. Some important components that contribute to student success once they are enrolled in the nursing program are resources such as tutoring, language resources for English as a second language students, culture guides (peer guides), faculty advisors, social and emotional support and financial support. It is also important to prepare these students for future leadership and encourage participation in student organizations. Students may also need assistance to create professional development plans or mind maps to help them envision themselves achieving higher level education and in leadership roles. Faculty pipeline programs should include pedagogical and socialization mentorship components and if tenure track, research support. 

## Research

Nursing education programs should identify creative ways to Increase the number of racially and ethnically diverse research scientists that represent the growing diversity of the United States. Development of pipeline programs early in undergraduate programs that focus on mentoring the next generation of researchers is essential to achieve this aim. Similarly, to other pipeline programs, it is also necessary to address barriers to graduate level education including support for entrance requirements, financial resources, and socialization to another possibly new environment.

There is a goal for increasing the number of minority researchers who have interest in addressing unsolved problems that disproportionately affect minority populations. It is more likely that minority researchers will create community participatory research, broaden the clinical and health services research agenda to include problems specific to minority populations, and develop culturally sensitive interventions which are customized for underrepresented populations^(^ 2 ^)^.

## Clinical

Clinical nursing workforce diversity is also multidimensional. Health systems must take initiative to connect with community partners to understand the needs and issues of importance within the community in order to better understand the resources which are needed to improve access to care. Returning to our social mission roots in community orientated health care delivery models and increasing nurse-managed primary care will help to increase access for vulnerable populations. 

The Institutes of Medicine (IOM) *Future of Nursing Report* (Institutes of Medicine, IOM, 2010^(^ 4 ^)^ recommendation is to make diversity in the nursing workforce a priority. The American Association of Colleges of Nursing and National League of Nursing have implemented strategies to address the IOM Campaign^(^ 4 ^)^. The Health Resources and Services Administration (HRSA), the primary federal agency that is tasked with improving access to health care services for the uninsured, isolated or medically vulnerable, has long recognized the importance of diversity and created nursing workforce diversity grant programs to enhance the understanding of barriers to achieving these goals and strategies to address. It takes support from these high-level organizations and from the institutional leadership to elevate the charge of increasing nursing workforce diversity. We all must address this initiative with fervor and dedication in order to have an impact on eliminating health disparities and achieving health equity.
